# Supplementary material for: Multiplexed live-cell profiling with Raman probes
Source: Nat Commun. 2021 Jun 7;12:3405. doi: 10.1038/s41467-021-23700-0 (PMC8184955; doi:10.1038/s41467-021-23700-0)
Supplement: Supplementary file 1 — Supplementary Information [file 41467_2021_23700_MOESM1_ESM.pdf]

Multiplexed Live-Cell Profiling with Raman probes

Chen Chen[1,2], Zhilun Zhao[1,2], Naixin Qian[1], Shixuan Wei[1], Fanghao Hu[1] and Wei Min[1,*]

[1]Department of Chemistry, Columbia University, New York, NY 10027

[2]These authors contributed equally: Chen Chen, Zhilun Zhao

*Corresponding Author: wm2256@columbia.edu

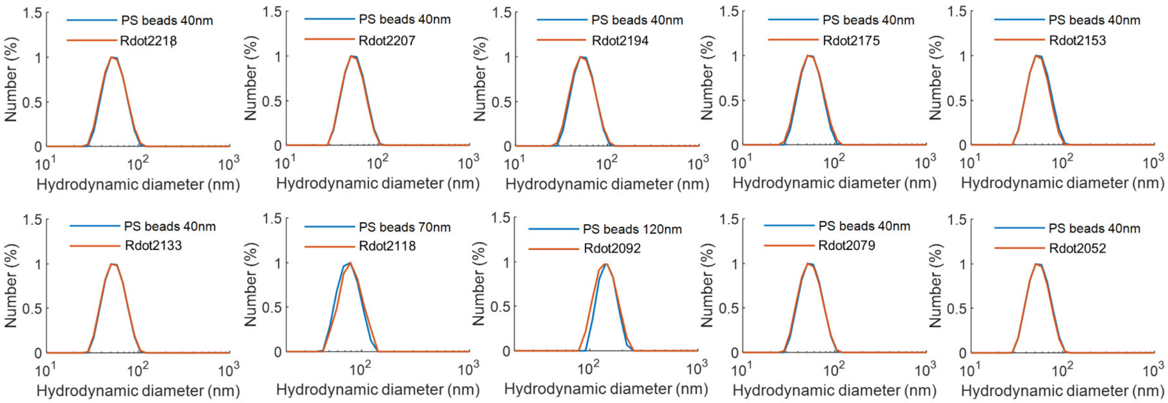

Supplementary Figure 1. DLS size characterization of ten-colored Rdots before and after dye doped.

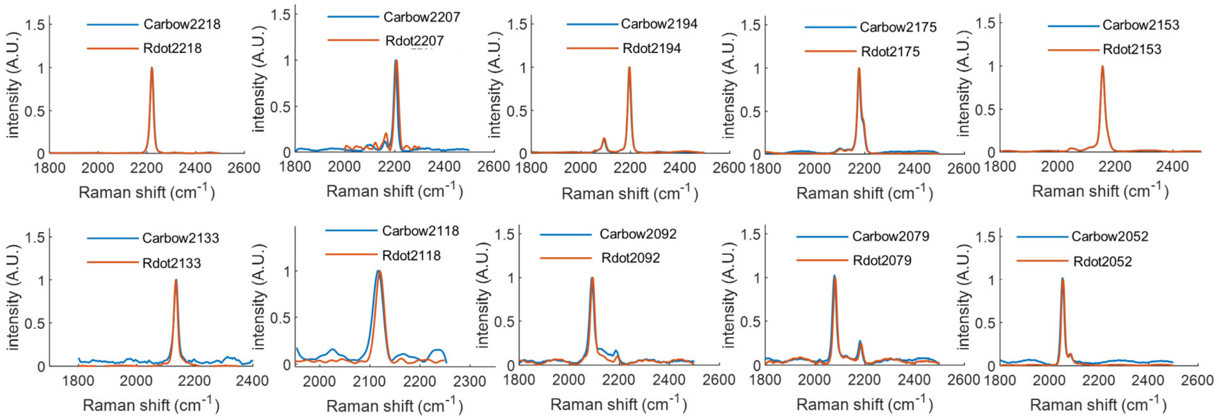

Supplementary Figure 2. Raman spectra of ten-colored Rdots in water and free Carbow dyes in DMSO. A.U.: arbitrary units.

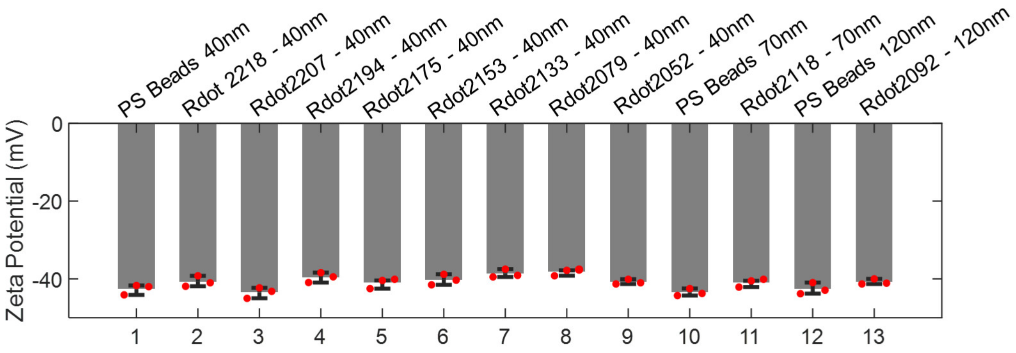

Supplementary Figure 3. DLS zeta potential characterization before and after dye doped. Error bars denote mean ± standard deviation, n=3. The PS beads are negatively charged with carboxyl groups functionalized. After the swelling-shrinking process, the surface carboxyl groups were mostly retained.

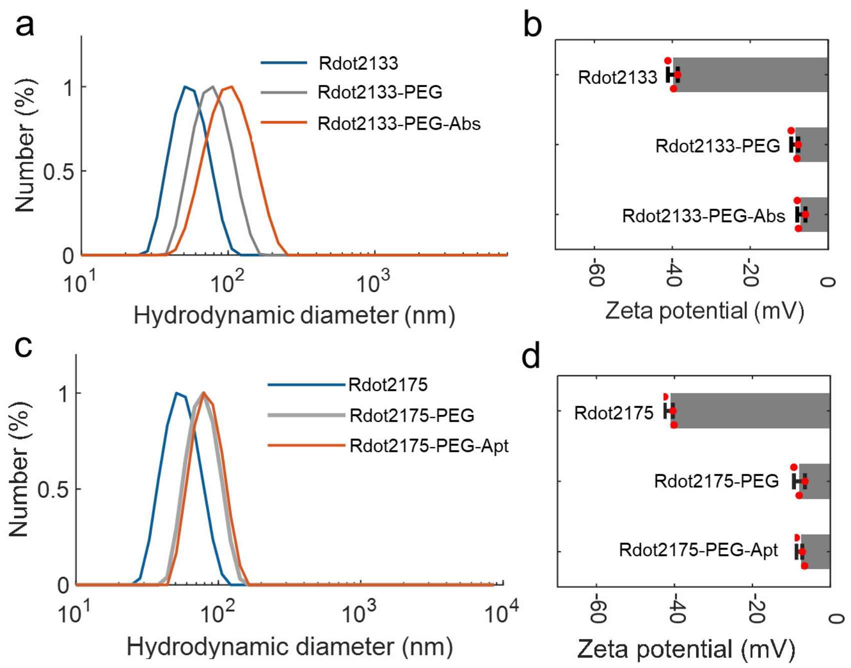

Supplementary Figure 4. The DLS characterization of Rdots conjugates. (a) Measured by DLS, the hydrodynamic diameter of Rdots increased by 20 nm after PEGylation. The surface antibody conjugation also induces an increase in size by approximately 40 nm. (b) Due to the replacement of a large proportion of surface carboxyl groups with amine-PEG-alcohol, The PEGylation yielded a change in zeta potential from -40 mV to -10 mV. Error bars denote mean $\pm$ standard deviation, n=3. (c) The hydrodynamic diameter of Rdots-aptamer conjugates is about 70 nm, much smaller than antibody-coated Rdots. (d) Zeta potential characterization of Rdots-aptamer conjugates. Error bars denote mean $\pm$ standard deviation, n=3.

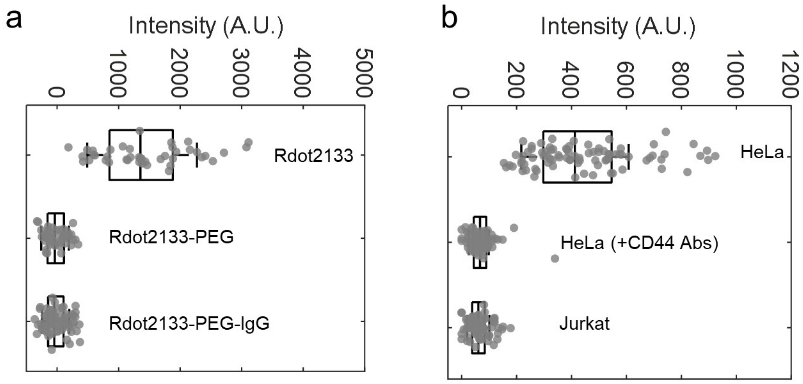

Supplementary Figure 5. Specificity test of Rdots conjugates. (a) The nonspecific binding was evaluated by Raman intensity measurements of cells mixed with Rdots at working concentration. An obvious Raman signal was detected for cells incubated with carboxylated Rdots, while cells incubated with PEGylated Rdots exhibited little Raman intensity. Consequently, PEG functionalization yields a passivated surface that minimizes cellular interaction by almost 90%. By conjugating PEGylated Rdots with IgG that lacks specificity to the target, we tested the nonspecific binding of the negative control sample. As expected, negligible staining was observed, validating no obvious background staining aroused during the introducing of target molecules. $N_{Rdot2133} = 42$; $N_{Rdot2133\text{-}PEG} = 43$; $N_{Rdot2133\text{-}PEG\text{-}IgG} = 82$. (b) Rdot2218-CD44 stained HeLa cell exhibits a strong Raman signal, while both the pre-blocked HeLa cells and the CD44-negative cell line Jurkat produce nearly no Raman signal, validating the targeting specificity of Rdots conjugates. Box edges indicate the interquartile range (IQR); center line, median; whiskers, lowest and highest data within 0.5IQR from the first and third quartiles, respectively. $N_{HeLa} = 85$; $N_{HeLa\ (+CD44)} = 74$; $N_{Jurkat} = 75$. A.U.: arbitrary units.

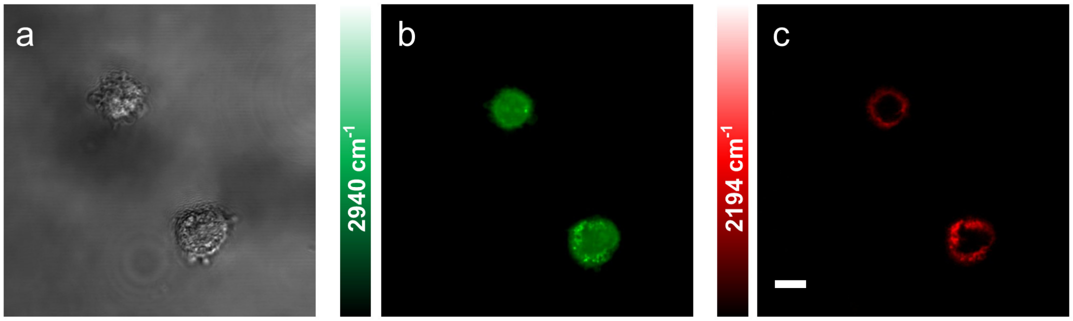

Supplementary Figure 6. Bright-field image (a), SRS image of frequency of 2940 cm^{-1} (b) and 2194 cm^{-1} (c) of Rdot2194-EpCAM positively stained HeLa cells, scale bar: 10 μm. Indicated by the bright field and CH_3 protein channels at 2940 cm^{-1}, most Rdots well located on the cell membrane surface, confirming the specific recognition. The color scales display relative SRS signal level. Image results were representative of three cell replicates.

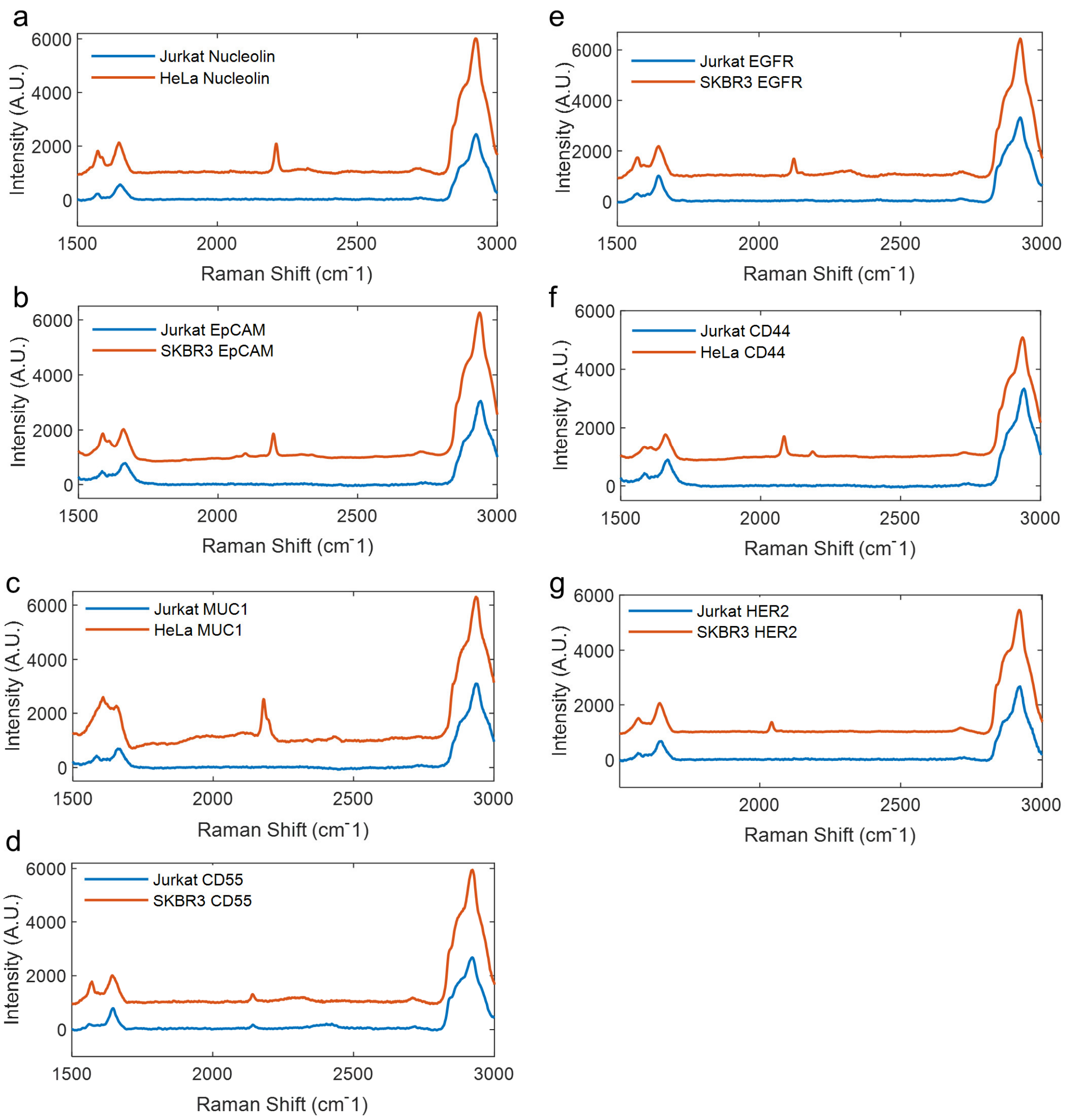

Supplementary Figure 7. The specificity and semi-quantitative measurement of seven-colored Rdots conjugates. Cells were stained with targeted Rdots conjugates, respectively. (a) Rdot2218-Nucleolin stained positive cell HeLa and negative cell Jurkat, averaged over 105 and 88 cells, respectively; (b) Rdot2194-EpCAM stained positive cell SKBR3 and negative cell Jurkat, averaged over 112 and 140 cells, respectively; (c) Rdot2175-MUC1 stained positive cell HeLa and negative cell Jurkat, averaged over 75 and 80 cells, respectively; (d) Rdot2153-CD55 stained SKBR3 and Jurkat cells with moderate expression, averaged over 155 and 77 cells, respectively. According to the human protein atlas database, the normalized expression of CD55 is 1.5 for Jurkat cell and 10.1 for SKBR3 cell. Consistent with the expression levels reported, the peak intensity of SKBR3 cells is about 3 times larger than Jurkat cells, indicating a linear correlation between the Raman intensity and expression level. (e) Rdot2133-EGFR stained positive cell SKBR3 and negative cell Jurkat, averaged over 151 and 140 cells, respectively; (f) Rdot2079-CD44 stained positive cell HeLa and negative cell Jurkat, averaged over 124 and 83 cells, respectively; (g) Rdot2052- HER2 stained positive cell SKBR3 and negative cell Jurkat, averaged over 92 and 86 cells, respectively. A.U.: arbitrary units.

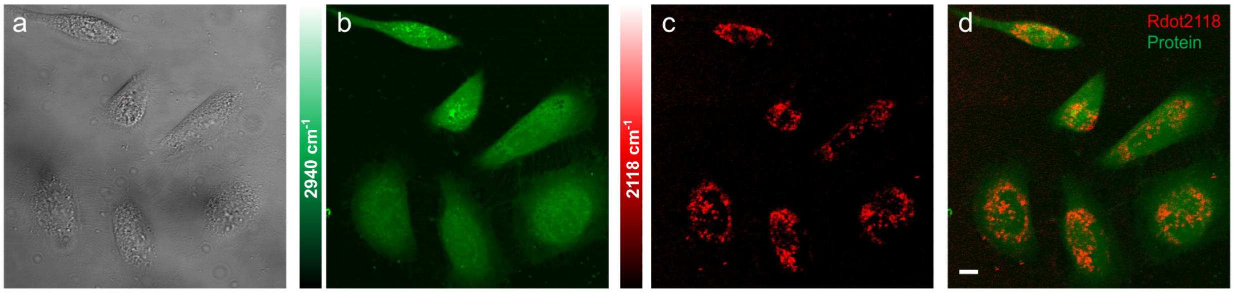

Supplementary Figure 8. Bright-field image (a), SRS image of cell protein (b), 70 nm Rdots (c) and merged channel (d) of HeLa cells incubated with 70 nm Rdots for 6 hours, scale bar: 10 μm. Indicated by the bright field and protein channels at 2940 cm^{-1}, most Rdots tend to accumulate in the perinuclear region. The color scales display relative SRS signal level. Image results were representative of four cell replicates.

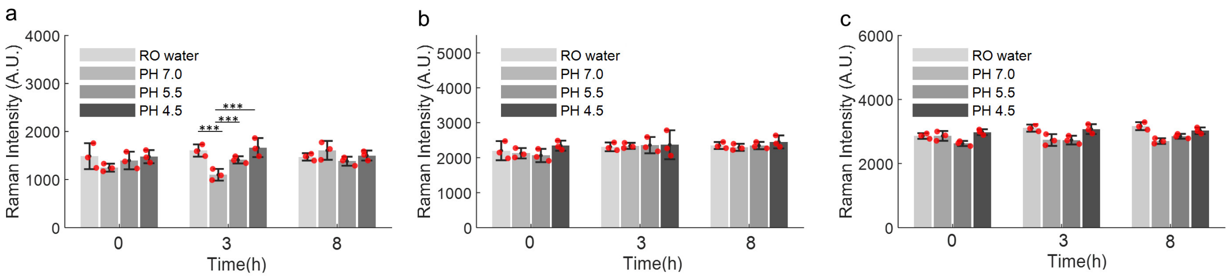

Supplementary Figure 9. PH stability of Rdots in the cellular microenvironment. Bar plots illustrating the time-lapse Raman intensity stability of endocytic Rdots at pH 4.5, pH 5.5, pH 7.0 and RO water (a) 40 nm endocytic Rdot2207; (b) 70 nm endocytic Rdot2118; (c) 120 nm endocytic Rdot2092. The error bar indicates mean ± standard deviation. ***denotes a significant difference (P<0.001, two-tailed). No significant difference observed for the non-denoted groups. A.U.: arbitrary units.

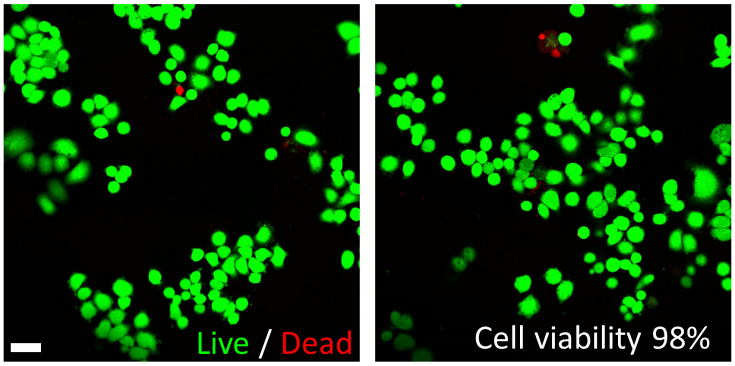

Supplementary Figure 10. Cytotoxicity assay of Rdots on cells. After surface protein labeling and endocytic test with Rdots, live SKBR3 cells were stained with calcein-AM (green) and dead SKBR3 cells stained with PI (red), scale bar: 40 μm. Image results were representative of two cell replicates.

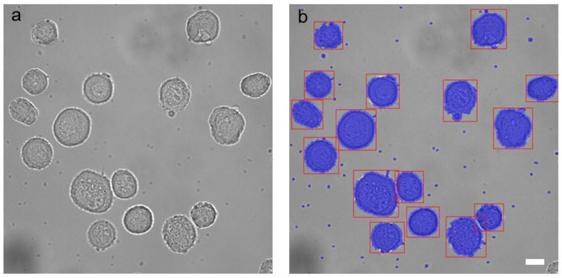

Supplementary Figure 11. High cell recognition rate of home-built Raman microscope (a) bright field of view (b) cell identification and localization results. Scale bar: 5 μm. Image results were representative of two cell replicates.

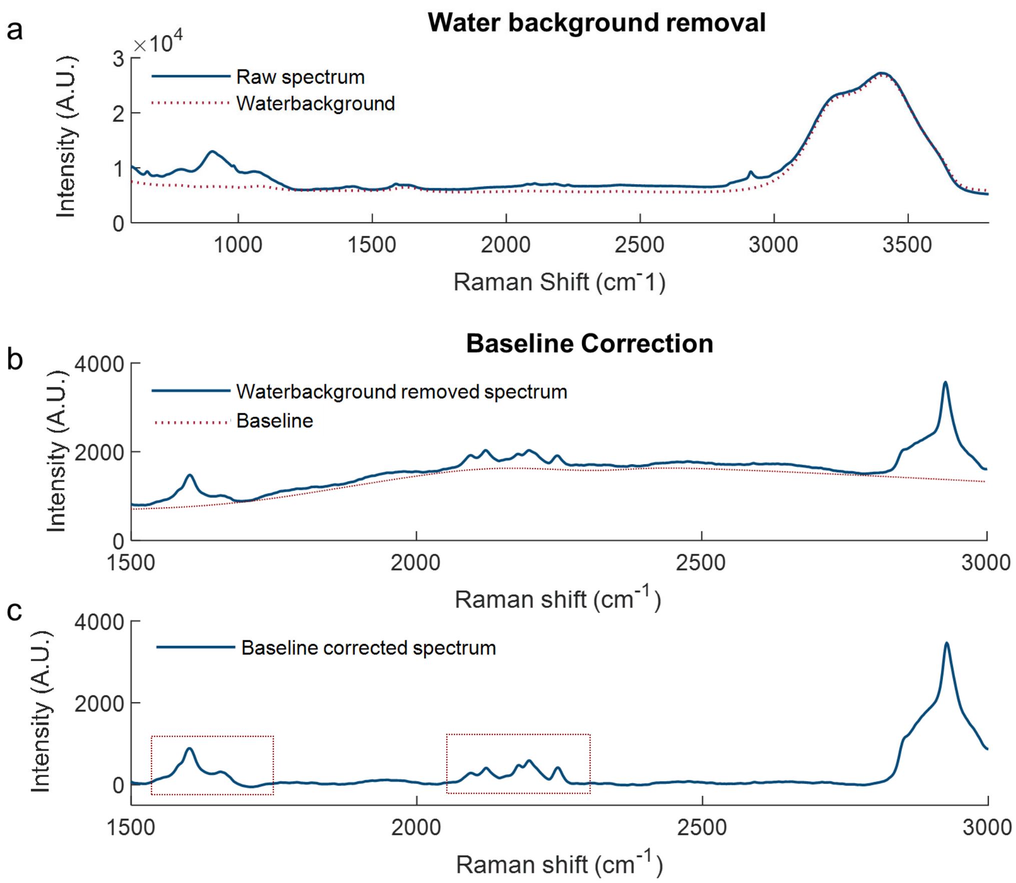

Supplementary Figure 12. Spectral preprocessing procedures (a) Water background removal (b) Baseline correction (c) Baseline corrected spectrum. A.U.: arbitrary units.

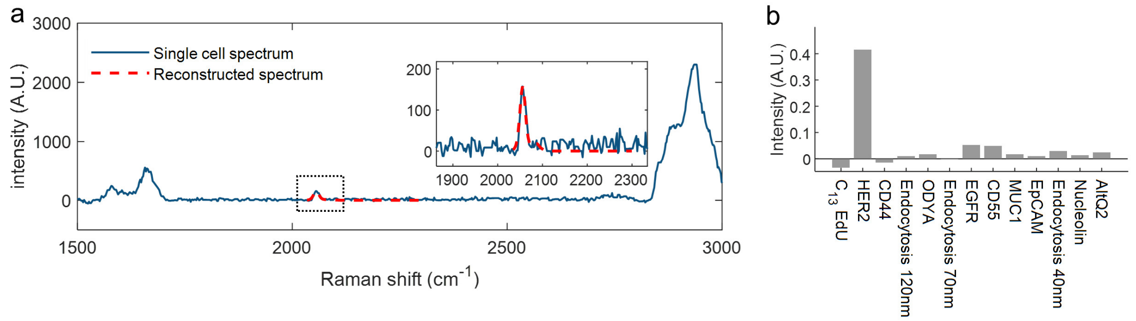

Supplementary Figure 13. Accuracy validation of spectral unmixing algorithm (a) Spectral unmixing of single-cell acquisition with low SNR. (b) Intensity distribution of each parameter based on unmixing results. A.U.: arbitrary units.

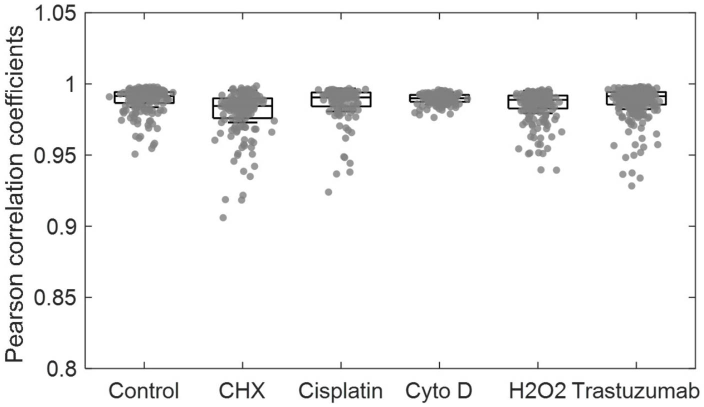

Supplementary Figure 14. The Pearson correlation coefficients distribution between reconstructed and raw single-cell spectra. The high correlation value (larger than 0.988 mostly) validates the robustness of the unmixing model. Box edges indicate the interquartile range (IQR); center line, median; whiskers, lowest and highest data within 0.5IQR from the first and third quartiles, respectively. $N_{Control}$=175; N_{CytoD}=182; N_{CHX}=133; $N_{trastuzumab}$=246; $N_{Cisplatin}$=131; N_{H2O2}=145.

Supplementary Note 1: Labeling efficiency estimation of Rdots

To quantify the number of incorporated Rdots, we first need to measure the absolute Raman intensity of a single Rdot. The linear relationship between Raman intensity at 3055cm^{-1} (C–H vibration in styrene) and the volume of a single bead (Supplementary Figure 15) implies that the density of polystyrene (PS) beads at different sizes is identical. So we can take the 3 μm PS beads to estimate the Raman intensity of a single Rdot. The volume size of a single 3 μm bead equals to that of 4.2×10^5 Rdots at 40 nm. Since the Raman intensity of 3 μm PS bead at 3055 cm^{-1} is 7.8×10^3 counts/200 mW*4s (Supplementary Figure 16), the intensity of 3055 cm^{-1} peak for single 40 nm Rdot is $7.8\times 10^3/4.2\times 10^5 = 1.9 \times 10^{-2}$ counts/200 mW*4s, or 9.3×10^{-3} counts/400 mW*s.

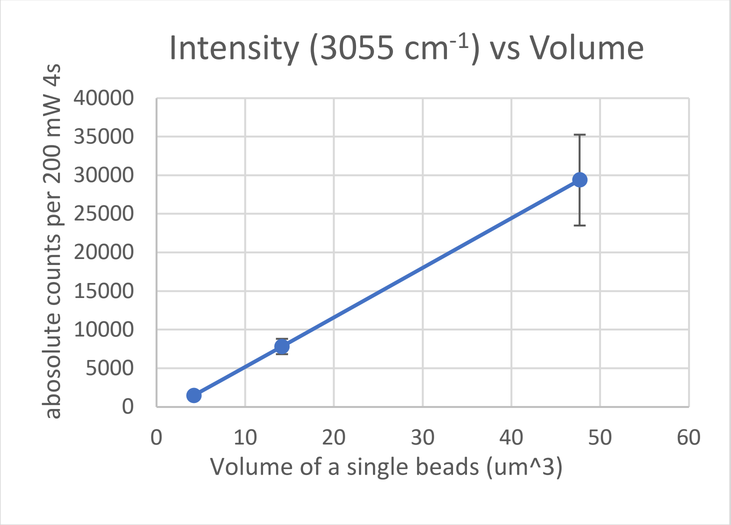

Supplementary Figure 15. The linear relationship between Raman intensity of 3055cm^{-1} and volume of a single bead. Error bars denote mean ± standard deviation, n=5 for each size.

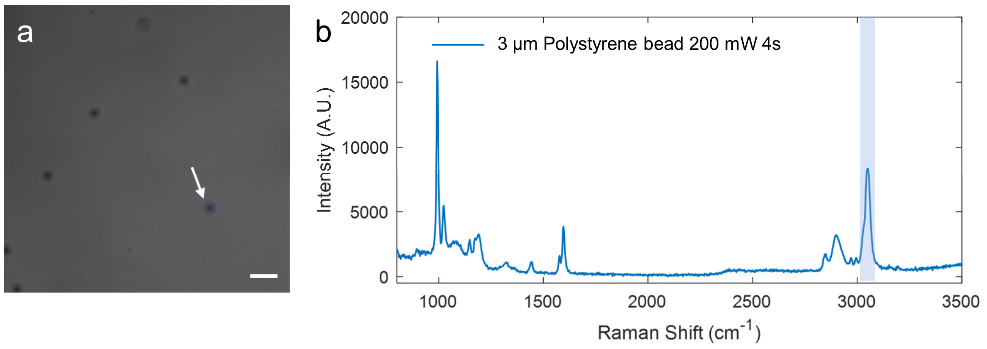

Supplementary Figure 16. (a) Bright-field image of suspended 3 μm polystyrene beads, (b) Raman spectrum of a single 3 μm polystyrene bead (indicated by the arrow in a). Excitation: 532 nm (200 mW), acquisition time: 4 s. Scale bar: 10 μm. Image results were representative of five bead replicates. A.U.: arbitrary units.

For Rdot2218, the Raman signal ratio of 2218 cm^{-1} peak (C≡C vibration in Carbow2218) and 3055 cm^{-1} peak is calculated: $R_{2218/3055} = 6.0$ (Supplementary Figure 17). So the intensity of 2218 cm^{-1} peak for single 40 nm Rdot is $9.3\times 10^{-3}\times 6.0 = 5.6 \times 10^{-2}$ counts/400 mW*s. As shown in Figure 2b in the manuscript, the absolute Raman intensity at 2218 $cm^{-1}$of Rdot2218-CD44 on the single HeLa cell is 500 counts/400 mW*s. As a result, the number of Rdot2218-CD44 on the single cell is $500/5.6 \times 10^{-2} = 9.0\times 10^{3}$. As reported, the abundance of CD44 protein in HeLa cells is about 2 million copies/cell[1]. Then we could estimate that the labeling efficiency of Rdot2218-CD44 on HeLa cell is about 4.5‰. Here, we provide a general approach to estimate the labeling efficiency of Rdots. This value greatly depends on the copy number and membrane assemblies of proteins and thus varies for different proteins and cell types.

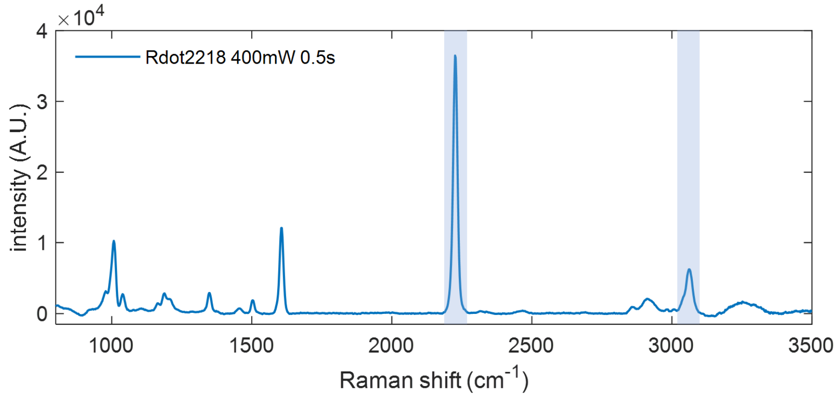

Supplementary Figure 17. Raman spectrum of 40 nm Rdot2218 solution. Excitation: 532 nm (400 mW), acquisition time: 0.5 s. A.U.: arbitrary units.

Supplementary Note 2: Quantification of the internalized endocytic Rdots

A similar calculation can be applied to quantify the number of internalized endocytic Rdots. Taking Rdot2092-120nm as an example, the volume size of a single 3 μm PS bead equals to that of 1.6×10^4 Rdots at 120 nm diameter. As a result, the intensity of 3055 cm^{-1} peak for a single 120 nm Rdot is 0.25 counts/400 mW*s. The Raman signal ratio of 2092 cm^{-1} peak and 3055 cm^{-1} peak is calculated: $R_{2092/3055} = 6.0$ (Supplementary Figure 18). So the intensity of 2092 cm^{-1} peak for a single 120 nm Rdot is 1.50 counts/400 mW*s. As shown in Figure 3a in the manuscript, the absolute Raman intensity of 2092 cm^{-1} peak acquired after incubated with endocytic Rdots for 6 hours is about 150 counts/400 mW*s, indicating the number of internalized Rdot2092 is 150/1.50=100. Under a similar calculation, the numbers of internalized Rdot2118-70nm and Rdot2207-40nm were 1300 and 9000 respectively.

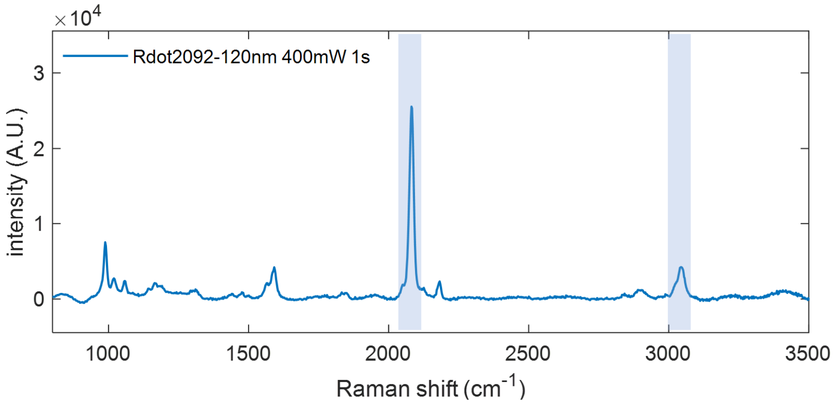

Supplementary Figure 18. Raman spectrum of 120 nm Rdot2092 solution. Excitation: 532 nm (400 mW), acquisition time: 1 s. A.U.: arbitrary units.

Supplementary Movie 1. A video clip showing the user interface of our home-built single-cell Raman profiling platform and demonstrating the automated process of acquiring Raman spectra from single cells.

Dye	Carbow 2218	Carbow 2207	Carbow 2194	Carbow 2175	Carbow 2153	Carbow 2133	Carbow 2118	Carbow 2092	Carbow 2079	Carbow 2052
Concentration	10 mM	10 mM	10 mM	2.5 mM	2.5 mM	1 mM	1 mM	1 mM	1 mM	1 mM

Supplementary Table 1. The concentration of incorporated Carbow dyes.

Antibody/ Aptamer	Catalog numbers/ sequences
Mouse anti-human CD55 Clone: 28	Invitrogen MA5-29118
Rat anti-human CD44 Clone: IM7	Invitrogen 14044185
Mouse anti-human EGFR Clone: 528	Bio X Cell BE0279R005MG
Nucleolin aptamer[2]	/5AmMC6/TTTTTTGGTGGTGGTGGTTGTGGTGGTGGTGG
MUC1 aptamer[3]	/5AmMC6/TTTTTTGCAGTTGATCCTTTGGATACCCTGG
HER2 aptamer[4]	/5AmMC6/TTTTTTAACCGCCCAAATCCCTAAGAGTCTGCACTTGTC ATTTTGTATATGTATTTGGTTTTTGGCTCTCACAGACACACTACAC ACGCACA
EpCAM aptamer[4]	/5AmMC6/TTTTTTCACTACAGAGGTTGCGTCTGTCCCACGTTGTCAT GGGGGGTTGGCCTG

Supplementary Table 2. Antibodies and aptamers used for cell surface protein staining.

Chemotherapy agents	Mechanism of action	Concentration
Cisplatin	DNA synthesis inhibitor	0.2 µg/mL
Cycloheximide	Protein synthesis inhibitor	1 µg/mL
Trastuzumab	Monoclonal antibody targeting HER2	100 ng/mL
Cytochalasin D	Actin inhibitor	2.5 µg/mL
H_2O_2	chemical oxidation of cellular components	100 µM

Supplementary Table 3. Chemotherapy reagents mechanism and concentration tested in this study.

Supplementary References

1. Itzhak, D. N.; Tyanova, S.; Cox, J.; Borner, G. H., Global, quantitative and dynamic mapping of protein subcellular localization. *Elife* **2016,** *5*.

2. Wang, J.; Liang, D.; Feng, J.; Tang, X., Multicolor Cocktail for Breast Cancer Multiplex Phenotype Targeting and Diagnosis Using Bioorthogonal Surface-Enhanced Raman Scattering Nanoprobes. *Anal Chem* **2019,** *91* (17), 11045-11054.

3. Nabavinia, M. S.; Gholoobi, A.; Charbgoo, F.; Nabavinia, M.; Ramezani, M.; Abnous, K., Anti-MUC1 aptamer: A potential opportunity for cancer treatment. *Med Res Rev* **2017,** *37* (6), 1518-1539.

4. Labib, M.; Green, B.; Mohamadi, R. M.; Mepham, A.; Ahmed, S. U.; Mahmoudian, L.; Chang, I. H.; Sargent, E. H.; Kelley, S. O., Aptamer and Antisense-Mediated Two-Dimensional Isolation of Specific Cancer Cell Subpopulations. *J Am Chem Soc* **2016,** *138* (8), 2476-9.
