# Supplementary material for: Multiplexed live-cell profiling with Raman probes
Source: Nat Commun. 2021 Jun 7;12:3405. doi: 10.1038/s41467-021-23700-0 (PMC8184955; doi:10.1038/s41467-021-23700-0)
Supplement: Supplementary file 2 — Reporting Summary [file 41467_2021_23700_MOESM2_ESM.pdf]

nature research

Corresponding author(s): Wei Min
Last updated by author(s): Apr 26, 2021

Reporting Summary

Nature Research wishes to improve the reproducibility of the work that we publish. This form provides structure for consistency and transparency in reporting. For further information on Nature Research policies, see our Editorial Policies and the Editorial Policy Checklist.

Statistics

For all statistical analyses, confirm that the following items are present in the figure legend, table legend, main text, or Methods section.

n/a	Confirmed	
☐	☒	The exact sample size (*n*) for each experimental group/condition, given as a discrete number and unit of measurement
☐	☒	A statement on whether measurements were taken from distinct samples or whether the same sample was measured repeatedly
☐	☒	The statistical test(s) used AND whether they are one- or two-sided<br>*Only common tests should be described solely by name; describe more complex techniques in the Methods section.*
☒	☐	A description of all covariates tested
☒	☐	A description of any assumptions or corrections, such as tests of normality and adjustment for multiple comparisons
☐	☒	A full description of the statistical parameters including central tendency (e.g. means) or other basic estimates (e.g. regression coefficient) AND variation (e.g. standard deviation) or associated estimates of uncertainty (e.g. confidence intervals)
☐	☒	For null hypothesis testing, the test statistic (e.g. *F*, *t*, *r*) with confidence intervals, effect sizes, degrees of freedom and *P* value noted<br>*Give P values as exact values whenever suitable.*
☒	☐	For Bayesian analysis, information on the choice of priors and Markov chain Monte Carlo settings
☒	☐	For hierarchical and complex designs, identification of the appropriate level for tests and full reporting of outcomes
☐	☒	Estimates of effect sizes (e.g. Cohen's *d*, Pearson's *r*), indicating how they were calculated

Our web collection on statistics for biologists contains articles on many of the points above.

Software and code

Policy information about availability of computer code

Data collection	Data were collected with Olympus software FV3000 and home-written LABVIEW (2017) program.
Data analysis	Data analysis was carried out with MATLAB R2019b and ImageJ v1.8.0. MATLAB codes are available at Github (doi.org/10.5281/zenodo.4707653).

For manuscripts utilizing custom algorithms or software that are central to the research but not yet described in published literature, software must be made available to editors and reviewers. We strongly encourage code deposition in a community repository (e.g. GitHub). See the Nature Research guidelines for submitting code & software for further information.

Data

Policy information about availability of data

All manuscripts must include a data availability statement. This statement should provide the following information, where applicable:

- Accession codes, unique identifiers, or web links for publicly available datasets
- A list of figures that have associated raw data
- A description of any restrictions on data availability

All relevant data supporting the findings of the study are available from the corresponding author upon request.

Field-specific reporting

Please select the one below that is the best fit for your research. If you are not sure, read the appropriate sections before making your selection.

☒ Life sciences ☐ Behavioural & social sciences ☐ Ecological, evolutionary & environmental sciences

For a reference copy of the document with all sections, see nature.com/documents/nr-reporting-summary-flat.pdf

Life sciences study design

All studies must disclose on these points even when the disclosure is negative.

Sample size	Experimental sample sizes are indicated in the figure captions. The sample size was determined based on the requirement to perform statistical tests and the availability of materials.
Data exclusions	For single-cell analysis, outlier single-cell acquisition data were excluded from analysis as demonstrated in the Method section.
Replication	The number of replicates is indicated in the manuscripts. All replications were successful.
Randomization	We do not have statistical model presented and no randomization is needed in the article.
Blinding	We do not have statistical model presented and no blinding is needed in the article.

Reporting for specific materials, systems and methods

We require information from authors about some types of materials, experimental systems and methods used in many studies. Here, indicate whether each material, system or method listed is relevant to your study. If you are not sure if a list item applies to your research, read the appropriate section before selecting a response.

Materials & experimental systems

n/a	Involved in the study
☐	☒ Antibodies
☐	☒ Eukaryotic cell lines
☒	☐ Palaeontology and archaeology
☒	☐ Animals and other organisms
☒	☐ Human research participants
☒	☐ Clinical data
☒	☐ Dual use research of concern

Methods

n/a	Involved in the study
☒	☐ ChIP-seq
☒	☐ Flow cytometry
☒	☐ MRI-based neuroimaging

Antibodies

Antibodies used	Mouse anti-human CD55 Clone: 28 (Invitrogen MA5-29118); Rat anti-human CD44 Clone: IM7 (Invitrogen 14044185); Mouse anti-human EGFR Clone: 528 (Bio X Cell BE0279).
Validation	Antibody was validated as described on the vendor's website.<br>According to the manufacturer, Invitrogen MA5-29118 has been successfully used in western blot, flow cytometry and immunofluorescent applications.<br>According to the manufacturer, Invitrogen 14044185 has been successfully used in western blot immunohistochemistry, immunoprecipitation, flow cytometry and immunofluorescent applications.<br>According to the manufacturer, Bio X Cell BE0279 has been successfully used in western blot immunohistochemistry, functional assays, immunoprecipitation, Immunohistochemistry, flow cytometry and immunofluorescent applications.

Eukaryotic cell lines

Policy information about cell lines

Cell line source(s)	HeLa, SKBR3 and COS-7 cells were all purchased from the American Type Culture Collection (ATCC)
Authentication	HeLa, SKBR3 and COS-7 cells were all authenticated by ATCC.
Mycoplasma contamination	Cell lines were tested negative for mycoplasma contamination.
Commonly misidentified lines<br>(See ICLAC register)	No commonly misidentified cell lines were used in the study
