# Supplementary material for: Multiplexed live-cell profiling with Raman probes
Source: Nat Commun. 2021 Jun 7;12:3405. doi: 10.1038/s41467-021-23700-0 (PMC8184955; doi:10.1038/s41467-021-23700-0)
Supplement: Supplementary file 5 — Description of Additional Supplementary Files [file 41467_2021_23700_MOESM5_ESM.pdf]

Description of Additional Supplementary Files

File Name: Supplementary Movie 1
Description: A video clip showing the user interface of our home-built single-cell Raman profiling platform and demonstrating the automated process of acquiring Raman spectra from single cells.
